# Engineering Stem Cells for Islet Replacement Therapy: Recent Advances and Barriers for Clinical Translation

**DOI:** 10.3390/cells15060532

**Published:** 2026-03-17

**Authors:** Jayachandra Kuncha, Sharmila Devi Veeraswamy, Carly M. Darden, Jeffrey Kirkland, Michael C. Lawrence, Juan S. Danobeitia, Bashoo Naziruddin

**Affiliations:** 1 Islet Cell Laboratory, Baylor Scott & White Research Institute, Dallas, TX 75246, USA; jayachandra.kuncha@bswhealth.org (J.K.); sharmilajayachandraphd@gmail.com (S.D.V.); michael.lawrence@bswehealth.org (M.C.L.); 2 Annette C. and Harold C. Simmons Transplant Institute, Baylor University Medical Center, Dallas, TX 75246, USA; carly.darden@bswhealth.org (C.M.D.); jeffrey.kirkland@bswhealth.org (J.K.); juan.danobeitia@bswhealth.org (J.S.D.)

**Keywords:** engineered stem cells, islet transplantation, islet replacement therapy, type 1 diabetes, type 2 diabetes

## Abstract

Diabetes mellitus remains a leading cause of morbidity worldwide, driven in type 1 diabetes by autoimmune destruction of pancreatic β-cells and in advanced type 2 diabetes by progressive β-cell dysfunction and failure. Diabetes affects around 830 million people globally, with the vast majority residing within low- and middle-income nations. Over the last few decades, the numbers of people who have diabetes and those with untreated diabetes have consistently increased. Although current pharmacologic therapies improve glycemic control, they do not restore functional β-cell mass. Consequently, strategies aimed at protecting, regenerating, or replacing insulin-producing cells have emerged as a major focus of regenerative medicine. Stem cell-based approaches offer the potential to generate renewable sources of glucose-responsive β-like cells, but challenges remain in achieving full functional maturation, immune protection, scalable manufacturing, and durable clinical engraftment. This review examines advances in engineering stem cell-derived insulin-producing cells for islet replacement therapy, with an emphasis on differentiation strategies, immunoprotective approaches, and the translational barriers that must be addressed for durable β-cell replacement.

## 1. Introduction

Pancreatic islets are multicellular endocrine clusters that maintain glucose homeostasis through coordinated secretion of insulin and glucagon. Type 1 diabetes (T1D) results from autoimmune destruction of β-cells, leading to absolute insulin deficiency, whereas advanced type 2 diabetes is characterized by progressive β-cell dysfunction superimposed on insulin resistance [1]. Clinical islet allotransplantation is a promising cell replacement therapy that has demonstrated that restoration of β-cell mass can improve glycemic control and reduce severe hypoglycemia in select T1D patients. However, durable graft function requires lifelong systemic immunosuppression, which carries risks of infection, toxicity, and other adverse effects.

Despite clinical proof of concept for β-cell replacement, islet transplantation remains limited by donor organ scarcity, immune-mediated graft rejection, and the toxicities associated with chronic immunosuppression. These constraints have driven the development of alternative strategies aimed at generating renewable, functional insulin-producing cells through stem cell engineering. Advances in pluripotent stem cell (PSC) differentiation, adult progenitor expansion, and bioengineered transplantation platforms now offer the potential to overcome many of the limitations inherent to donor-derived islet transplantation [2].

Insulin is the central regulator of systemic energy metabolism, coordinating glucose uptake, storage, and utilization across multiple tissues. In response to elevated blood glucose, pancreatic β-cells secrete insulin, promoting glucose uptake in muscle and adipose tissue while stimulating glycogen synthesis in the liver and suppressing lipolysis and proteolysis. Conversely, during hypoglycemia, insulin secretion decreases, and glucagon release from α cells counterbalances falling glucose levels to maintain metabolic homeostasis [3].

**Figure 1:**
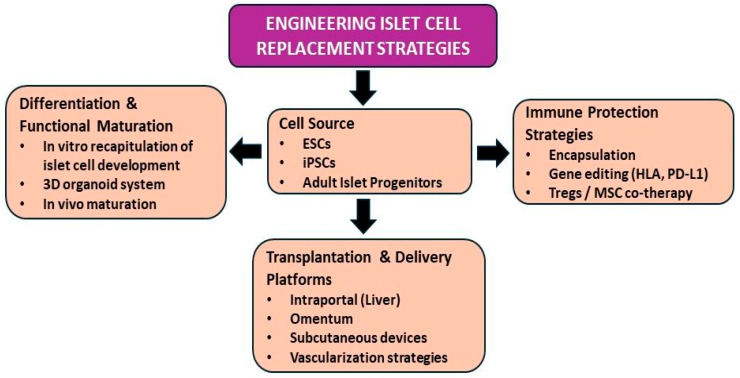
Conceptual framework of engineered islet cell replacement strategies. Stem cell-based islet replacement integrates four interconnected domains: (1) selection of appropriate cell sources, including pluripotent stem cells and adult pancreatic progenitor populations; (2) differentiation and functional maturation through in vitro recapitulation of islet cell development, 3D organoid systems, and in vivo maturation to establish multi-endocrine architecture; (3) immune protection strategies, including encapsulation and genetic immune engineering to mitigate allo- and autoimmune responses; and (4) optimization of transplantation and delivery platforms, such as intraportal, omental, and subcutaneous sites, to promote vascularization and durable engraftment.

Advances in diabetes technology, including continuous glucose monitoring and hybrid closed-loop insulin delivery systems, have significantly improved glycemic control and reduced hypoglycemia in many patients. However, even the most advanced artificial pancreas systems cannot fully replicate the real-time, cell-intrinsic sensing and pulsatile insulin secretion provided by endogenous β-cells, nor do they eliminate the long-term burden of device dependence and metabolic variability. Restoration of functional β-cell mass, therefore, remains a compelling therapeutic objective. Whole-organ pancreas transplantation achieves high rates of durable insulin independence in appropriately selected patients but is limited by surgical risk and donor availability. Islet transplantation offers a less invasive alternative and has demonstrated meaningful improvements in glycemic control and reduction in severe hypoglycemia, although long-term insulin independence rates are generally lower. Together, these clinical experiences validate β-cell replacement as a biologically effective strategy while underscoring limitations in scalability and immune durability. These constraints have driven intense efforts to engineer renewable insulin-producing cells and develop protective transplantation platforms capable of achieving durable engraftment (Figure 1).

In current clinical practice, β-cell replacement therapy is utilized not only for selected individuals with T1D but also in patients undergoing total pancreatectomy with islet autotransplantation for severe chronic pancreatitis. In this setting, autologous islets are infused intraportally to mitigate postoperative diabetes; however, many patients continue to require exogenous insulin due to limited islet mass and graft attrition. These limitations further underscore the need for renewable and functionally robust insulin-producing cell sources. Stem cell-based strategies aim to generate scalable, glucose-responsive β-like cells capable of overcoming donor scarcity and immune barriers inherent in traditional transplantation approaches.

In this review, we ask what the dominant rate-limiting steps to durable clinical engraftment of engineered islet grafts are and how do current stem cell and bioengineering platforms address them. We organize advances in cell source selection, differentiation and maturation, immunoprotection, and clinical translation around this central question, emphasizing where mechanistic progress has translated into durable function and where key barriers remain for widespread clinical adoption. Several recent reviews have considered stem cell-derived islets, encapsulation devices, or immune engineering separately. In contrast, we integrate these domains into a rate-limiting step framework centered on durable clinical engraftment. By comparing major platforms against shared translational endpoints intrinsic graft potency, long-term immune protection without chronic immunosuppression, safety, and scalable manufacturing. We aim to provide a critical, cross-platform synthesis rather than a purely descriptive overview.

## 2. Cell Sources, Scalability and Immunogenicity

### 2.1. Embryonic Stem Cells

Embryonic stem cells (ESCs) are pluripotent cells capable of differentiating into all somatic lineages, including pancreatic endocrine cells. Their unlimited proliferative capacity makes them an attractive source for generating insulin-producing cells at scale. However, clinical translation is constrained by concerns regarding teratoma formation, residual undifferentiated cells, and ethical considerations associated with embryo-derived cell lines [4,5]. Despite these challenges, ESCs have served as a foundational platform for the development of directed differentiation protocols aimed at producing functional β-like cells.

#### 2.1.1. Induced Pluripotent Stem Cells

Induced pluripotent stem cells (iPSCs) are generated through the reprogramming of somatic cells into a pluripotent state, thereby acquiring differentiation potential comparable to ESCs. iPSCs offer the advantage of patient-specific derivation, reducing ethical concerns and enabling potential autologous applications. Similar to ESCs, iPSCs possess extensive proliferative capacity, but concerns remain regarding genomic stability, epigenetic memory, and tumorigenicity following transplantation.

#### 2.1.2. Reprogramming Somatic Cells into Induced Pluripotent Stem Cells

Reprogramming of somatic cells into iPSCs is achieved through ectopic expression of defined pluripotency-associated transcription factors, enabling acquisition of ESC-like self-renewal and differentiation capacity. Advances in nonintegrating reprogramming methods have improved safety profiles and reduced the risk of insertional mutagenesis, enhancing translational feasibility.

#### 2.1.3. Advantages of Induced Pluripotent Stem Cells over Embryonic Stem Cells

iPSCs have significant benefits over ESCs. The iPSCs are derived from adult somatic cells, avoiding ethical concerns associated with ESCs. They can be generated from a patient’s own cells, reducing the risk of immune rejection in therapies. Additionally, iPSCs can be created in large quantities, enabling personalized medicine and disease modeling. They also hold promise for regenerative medicine and drug testing, offering a versatile and ethically more acceptable alternative to ESCs in research and clinical applications [6]. iPSCs possess the ability to self-renew and differentiate into various cell types, similar to ESCs. However, they can be patient-specific, offering the potential for autologous therapies and reduced immune rejection risks. Furthermore, iPSCs facilitate personalized medicine, allowing the study of genetic diseases and drug responses in vitro [7,8]. Nevertheless, autologous iPSC-derived β-like cells would remain susceptible to autoimmune recurrence in T1D, and large-scale manufacturing challenges are comparable to those encountered with ESC-based platforms. Consequently, both ESC and iPSC systems are being pursued in parallel for the generation of stem cell-derived islet cells.

### 2.2. Adult Stem Cells

Adult stem cells are tissue-resident progenitor populations that typically exhibit multipotent differentiation capacity and contribute to tissue maintenance and repair. Unlike PSCs, adult stem cells are generally lineage-restricted, limiting their differentiation potential but potentially reducing tumorigenic risk. In the context of islet regeneration, adult-derived cell populations have been investigated both as supportive immunomodulatory cells and as potential sources of pancreatic endocrine progenitors. Mesenchymal stem/stromal cells (MSCs) have been explored in islet transplantation primarily for their immunomodulatory, anti-inflammatory, and pro-angiogenic properties. Co-transplantation of MSCs with islets may enhance graft survival and vascularization; however, MSCs themselves have not demonstrated robust or reproducible differentiation into functional insulin-producing cells. Hematopoietic stem cell-based immune modulation strategies have also been investigated in autoimmune diabetes but do not directly address β-cell replacement. In contrast to broadly multipotent adult stem cells, increasing attention has focused on pancreas-derived progenitor populations that retain endocrine lineage potential. Such populations may offer a tissue-specific alternative to PSC platforms for generating functional islet-like structures.

## 3. Differentiation Engineering Towards Clinically Competent β-like Cells

**Figure 2:**
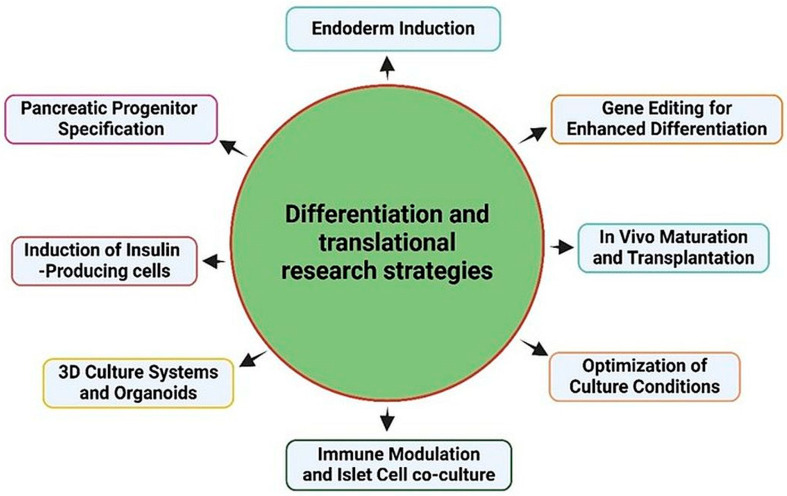
Differentiation and translational strategies used for the generation of islet-like cells to treat diabetes.

Stem cells can be differentiated into islet-like cells and may be used for β-cell replacement therapy for T1D. Several strategies, such as using PSCs, act similarly to the natural developmental processes of pancreatic islet cells. Based on the environmental factors that promote differentiation and maturation, differentiation strategies are used in stem cell engineering for islet replacement therapy. The mechanism of pancreatic endoderm induction is significant, as it distinguishes the pancreatic lineage from that of other organs that emerge from the endoderm. Diabetes therapeutics are being developed using stem cell-based techniques that mimic the process of pancreatic endoderm development (Figure 2).

Activin, bone morphogenetic protein, or retinoic acid signaling is sufficient to stimulate PDX1 expression in the endoderm before the pancreas. The lateral plate mesoderm appears to pattern the endoderm in a posterior-dominant manner, as seen previously in neural tube design at the same embryonic phase. These results support the mesoderm’s important function in coordinating the A-P pattern throughout all three major germ cells [9,10,11].

To obtain insulin-producing cells, various methods have been used, including impulsive differentiation with the selection of Nestin + progenitor cells [12]; restriction of phosphatidylinositol-3-kinase [13]; mimicking of the in vivo development process by incorporating differentiation components [14,15] during co-culture with embryonic pancreatic buds or culture in the presence of fetal pancreas-conditioned medium; and use of transgenic levels of pancreas-specific transcription factors, like neuroD1, ngn3, pax4, NKX6.1, foxa2, ptf1a, and PDX1 [12,16,17].

**Figure 3:**
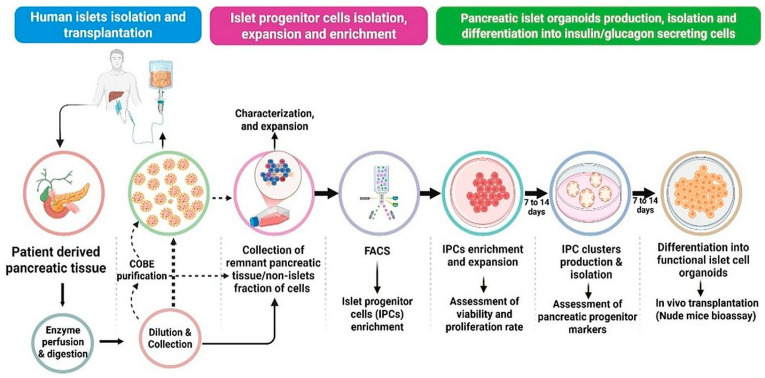
Key steps in the study, from patient-derived pancreatic tissue collection through progenitor cell isolation, expansion, differentiation into islet cell organoids, molecular characterization, and functional assessment.

Our research team conducted a thorough study to isolate and expand patient-derived multipotent adult islet progenitor cells. Our goal was to generate functional islet cell organoids in vitro, creating a renewable source of tissue for islet cell replacement therapy (Figure 3). To achieve this, we used non-islet recombination, wash, and COBE fractions obtained during good manufacturing practice-compliant isolation procedures from patients undergoing total pancreatectomy with islet autotransplantation. We isolated adult islet progenitors through flow-activated cell sorting and expanded them to clonal density, which allowed for the formation of organoids. We then differentiated them into mature islet cell organoids by culturing them in differentiation medium for 2 to 14 days. We characterized the resulting islet cell organoids using 10× Genomics single-cell transcriptomics, along with assessments of islet endocrine markers and functional properties. Single-cell RNA sequencing showed that organoids from adult islet progenitor cells became transcriptionally similar to islet cells isolated from cadaveric donors within 8 to 10 days of differentiation. Quantitative polymerase chain reaction validation confirmed strong expression of key α- and β-cell-specific transcription factors such as PDX1, NKX2.2, MafA, MafB, Pax6, Arx, and NKX6.1. We also observed markers of β-cell maturation, including glucokinase, GLUT2, proprotein convertase 1/3, and chromogranin A. Functionally, fully differentiated islet cell organoids consisted of mono-hormonal endocrine cells that showed strong glucose-responsive hormone secretion in a static glucose-stimulated insulin secretion (GSIS) assay. They released insulin in response to high glucose levels and glucagon in response to low glucose levels, under the specific in vitro conditions tested. Although donor variability, and long-term stability were not established. These results suggest at least comparable functional capacity in vitro but do not yet establish superiority over native islets in vivo. These findings show that we can successfully harvest multipotent islet progenitor cells from patient-derived pancreatic tissue and differentiate them into islet cell organoids with functional features similar to those of native human islets. This new method offers budding future for providing an unlimited source of patient-specific islet organoid tissue, which could either replace lost islet cells or support autologous islet transplantation after pancreatectomy [18,19].

These findings demonstrate that adult islet progenitor-derived organoids can acquire transcriptional and functional features of human islets in vitro and represent a promising autologous or lineage restricted platform. However, compared with pluripotent stem cell-derived islets, adult progenitor strategies currently have more limited manufacturing experience and are most realistically suited to niche indications such as TPIAT rather than broad allogeneic deployment. Direct head-to-head comparisons with PSC-derived products in relevant in vivo models will be required to define their specific translational significance.

A 3D culture system is useful for cellular expansion and differentiation, as well as for simulating the natural environment. It causes identical islet gene expression profiles throughout differentiation processes, but it greatly lowers cell loss and increases the survival of endocrine clusters. Orbital shaker-based suspension culture techniques have also been widely used to differentiate human PSC-derived pancreatic progenitors into islet-like clusters during endocrine induction stages. Yet reproducibility between experiments is impeded by varying degrees of cell loss in shaking cultures, resulting in varied differentiation rates. The static culture approach produces more repeatable and efficient human PSC islets that respond to glucose and secrete insulin. The successful differentiation and well-to-well consistency in 96-well plates demonstrate that the static 3D culture method can be used as a platform for small-scale compound screening research while also facilitating additional procedural improvements [20,21].

The induction of insulin-producing cells is usually accomplished by influencing stem cells or progenitor cells in a laboratory environment, employing specific chemical stimuli like activins, retinoic acid, or nicotinamide to direct their differentiation into functioning β-cells that manage insulin production in the pancreas [22]. Scientists frequently utilize ESCs or iPSCs as foundational materials for creating insulin-producing cells. Activin A, nicotinamide, exendin-4, and retinoic acid are commonly used in the growth medium to guide the differentiation of the stem cells into β-like cells [23,24].

CRISPR-Cas9 (clustered regularly interspaced short palindromic repeats–CRISPR-associated protein 9) is an emerging gene-editing tool that can be utilized to transform other cells into insulin-producing cells [25]. This technology enables scientists to precisely change the genetic information within these cells, which could facilitate the advancement of therapies for T1D by either generating functioning insulin-producing cells from stem cells or simply altering remaining β-cells to enhance their function. This is frequently accomplished by activating or inhibiting specific genes associated with insulin production and immune evasion to avoid rejection following transplantation. CRISPR is used to modify the genes of PSCs, directing them to develop into insulin-producing β-cells that can be implanted into individuals with diabetes [26]. It also corrects genetic abnormalities in preexisting β-cells that cause poor insulin production, potentially restoring their normal function. Investigators may be able to design β-cells to be less immunogenic, thus lowering the likelihood of rejection following transplantation in patients with T1D [27,28].

To improve the functionality of differentiated insulin-producing cells, co-culture techniques are currently being used, in which stem cell-derived β-cells are grown with other islet cell types such as α and δ cells [29]. Combining β-cells with α cells in predetermined ratios can enhance β-cell functioning by improving glucose sensing and the release of insulin [30]. To reduce immunological rejection following transplantation, some treatments use genetic modification to establish immunological tolerance in differentiated islet cells. This includes removing major histocompatibility complex genes, which may expose these cells to immunological assault [31,32].

One of the most challenging aspects of in vivo maturation and transplantation is ensuring that differentiated cells reach complete maturation prior to transplantation. As a result, some investigators prefer in vivo maturation, in which stem cell-derived cells are implanted into an animal model to undergo additional maturation to attain capacities of modified stem cell-derived islet-like cells. Transplanting stem cell-derived progenitors into animal models like mice or pigs allows the cells to develop within a living organism, which is comparable to how β-cells mature in genuine pancreatic islets. After transplantation into diabetic mice, in vivo maturation can enhance cell functionality by increasing insulin secretion and glucose-detecting capabilities [33,34].

Optimizing an appropriate nutrient-rich medium, including key nutrients such as amino acids, vitamins, and growth factors, can aid in the successful differentiation of engineered stem cells into insulin-producing cells. The culture conditions, such as oxygen levels, nourishment, and cell density, have a substantial impact on differentiation. Researchers modified these settings to boost differentiation efficiency and β-cell activity suitable for clinical application [35,36].

Across protocols, a key trade off emerges between fully differentiated β-like cells and implantation of pancreatic progenitors. Fully differentiated products offer more predictable phenotypes at the time of implantation but may remain functionally immature, whereas progenitor implantation leverages in vivo cues for final maturation at the cost of less control and potentially greater heterogeneity. Static 3D cultures improve reproducibility and are amenable to small scale screening, while suspension systems enable scale-up but can suffer from variable cluster loss and differentiation efficiency. These differences have direct implications for scalability, safety, and batch-to-batch consistency in a clinical manufacturing context.

A key unresolved question is whether optimal diabetes cell therapy requires reconstitution of islet-like organoids containing multiple endocrine cell types and native-like architecture, or whether β-cell-enriched preparations are sufficient. PSC-based protocols increasingly generate 3D clusters that include α and δ cells, with evidence that paracrine glucagon and somatostatin signaling can enhance glucose sensing and dynamic insulin secretion. By contrast, some differentiation approaches and many adult progenitor strategies tend to yield more β-cell dominant populations. While organoid-like islet structures may better recapitulate physiology, they also introduce additional complexity in manufacturing and quality control. Direct head-to-head comparisons of multi-endocrine organoids versus β-cell focused products on clinically relevant models are needed to determine how much added benefit full islet organization provides for long-term glycemic control and safety.

## 4. Functional Maturation of Engineered β-like Cells

Achieving functional maturity remains a central challenge in engineered β-cell replacement. Although directed differentiation protocols generate insulin-producing cells expressing canonical β-cell transcription factors, these cells frequently exhibit immature transcriptional profiles and suboptimal GSIS compared to adult human islets.

### 4.1. In Vitro Maturation

In vitro maturation strategies focus on optimizing culture architecture, metabolic conditioning, and endocrine cell composition. Three-dimensional organoid systems enhance cell–cell interactions and improve endocrine specification relative to two-dimensional culture. Adjustments in nutrient composition, oxygen tension, extracellular matrix scaffolding, and endocrine cluster size have all been shown to improve insulin content and GSIS dynamics. Incorporation of additional endocrine cell types, including α and δ cells, may further promote physiologic glucose responsiveness through the restoration of paracrine signaling networks.

### 4.2. In Vivo Maturation

Transplantation into immunodeficient animal models consistently enhances functional maturation. Exposure to physiologic vascularization, extracellular matrix cues, and systemic metabolic regulation improves insulin secretion kinetics and dynamic glucose responsiveness. These findings suggest that complete functional competency may require a combination of engineered in vitro differentiation and in vivo conditioning.

In vitro maturation strategies (3D cell culture, organoids, optimized media, and co-culture with α and δ cells) have improved insulin content and GSIS dynamics but largely remain unable to fully recapitulate adult human islet function. In vivo maturation following implantation into immunodeficient models further enhances dynamic glucose responsiveness yet introduces additional variables related to transplantation site and host physiology. A major unmet need is the definition of standardized phenotypic and functional benchmarks that predict long-term insulin independence in humans and allow rigorous comparison between in vitro and in vivo maturation-based platforms.

### 4.3. Transcriptomic and Functional Maturity Gaps

Despite progress, stem cell-derived β-like cells often retain partial fetal-like transcriptional signatures. Continued refinement of differentiation timing, endocrine cluster composition, and metabolic programming will be necessary to bridge the remaining maturity gap between engineered grafts and native adult islets.

## 5. Engineering Durable Immune Protection Beyond Systemic Immunosuppression

### 5.1. Precision Engineering of Islet Surfaces to Suppress Acute Inflammatory and Autoimmune Responses

Durable β-cell replacement requires protection from both alloimmune rejection and recurrent autoimmunity. As shown in islet transplantation reports, the stem cell-derived islet-like cells will also be subjected to innate immune responses [37]. Our team has identified toll-like receptor-4 as a major mediator of inflammatory response against transplanted islets [38]. We evaluated the efficacy of the TLR4 selective inhibitor TAK-242 to suppress innate and adaptive responses to allogeneic islets. Our data provided evidence that blockade of TLR4 signaling in both islets and local innate immune cells can suppress the acute inflammatory response and downstream proliferation and activation of CD8-positive T cells in an in vitro mixed lymphocyte reaction with islets and peripheral blood mononuclear cells [39]. We recently used click-chemistry to create a TAK-242-eluting islet cell product that could suppress inflammation and improve islet viability and function in an autologous model [38]. Our method of nanocoating TAK-242 on the cell surface does not increase the transplantable islet volume and retains cell viability and function for a prolonged period. Engineering strategies now extend beyond systemic immunosuppression to include biomaterial encapsulation and genetic immune modulation.

Our TLR4 targeted nanocoating illustrates how surface directed anti-inflammatory strategies can be layered onto both donor-derived and stem cell-derived islets without increasing implant volume. Nonetheless, these data remain preclinical, and the relative benefits of local TLR4 blockade versus systemic immunosuppression or alternative innate immune targets will need to be established in standardized comparative studies.

### 5.2. Encapsulation Approaches

**Figure 4:**
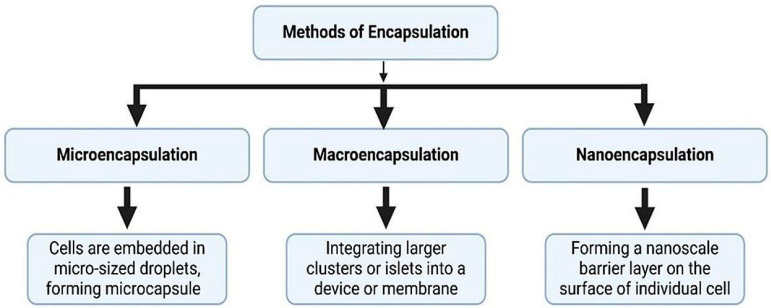
Different types of encapsulation methods are used for islet and engineered stem cells for protection against immunological rejection while preserving their function.

Encapsulation platforms aim to provide physical immune isolation while permitting oxygen and nutrient exchange (Figure 4). Microencapsulation encloses individual islet clusters within semipermeable hydrogels. Macroencapsulation devices house larger cell populations within retrievable implantable scaffolds. Nano-engineered coatings seek to reduce inflammatory activation and fibrotic overgrowth. While encapsulation may reduce dependence on systemic immunosuppression, challenges persist regarding long-term oxygen diffusion, fibrosis, and sustaining endocrine function.

### 5.3. Genetic Immune Engineering

Genome editing technologies such as CRISPR-Cas systems have enabled targeted modulation of immune recognition pathways. Strategies include HLA class I/II modification to reduce alloimmune detection, overexpression of immune checkpoint regulators such as PD-L1, engineering of hypoimmunogenic stem cell lines, and cytokine pathway modulation to reduce inflammatory signaling. Balancing immune evasion with oncologic safety remains critical.

Device-based immune isolation (macro, micro and nano-encapsulation, conformal coatings) offers the advantages of physical retrievability and containment of graft-related adverse events. Still, it is limited by diffusion constraints, fibrosis, and challenges in scaling cell dose. Genetic hypoimmunogenic engineering directly reduces allogeneic and autologous recognition and may ultimately obviate chronic immunosuppression, yet it raises concerns about infection surveillance, oncologic risk, and irretrievability once cells engraft. These complementary approaches are at different stages of validation, with encapsulation tested in early clinical trials and most hypoimmune cells remaining at the preclinical or early stages of clinical trials.

### 5.4. Immune Tolerance Induction

Since patients with T1D have preexisting autoimmunity and will develop an allogenic response post-transplant, inducing immune tolerance is another key strategy for improving the success of cell transplantation. Instead of relying on encapsulation or immunosuppressive drugs, immune tolerance induction aims to “train” the recipient’s immune system to accept the transplanted cells as self, preventing rejection [40].

### 5.5. Approaches to Induce Immune Tolerance

Adjunct immunomodulatory strategies aim to create a permissive microenvironment for graft survival. Approaches include regulatory T cell (Treg) therapy, MSC co-transplantation, and hematopoietic chimerism induction. Although promising, these methods require further validation for durability and safety in clinical settings. Among tolerance induction strategies, Treg therapies and MSC co-transplantation have demonstrated the most consistent capacity to modulate allogeneic and autoimmunity in rodent models, with emerging but still limited experience in human islet and solid organ transplantation. Mixed chimerism can achieve robust tolerance but requires intensive conditioning and carries significant procedural risk, limiting its applicability to routine T1D therapy. Key barriers to clinical deployment include scalability of cell-based immunotherapies, durability of tolerance once adjunctive immunosuppression is withdrawn, and the risk of generalized immunosuppression or off-target immune effects.

## 6. Transplantation Site Considerations

The choice of implantation site significantly influences engraftment efficiency, vascularization, and long-term function. The intraportal hepatic route remains the most established clinical approach, although it is associated with instant blood-mediated inflammatory reactions and limited graft retrievability. Alternative sites under investigation include the omentum, subcutaneous space with vascularizing scaffolds, and preperitoneal compartments. Emerging strategies integrate biomaterial scaffolds to enhance oxygen delivery and vascular integration across diverse anatomical sites. An optimal site must balance vascular access, immune monitoring, mechanical stability, and retrievability.

## 7. Preclinical Models of Engineered Islet Therapy

Preclinical models remain essential for evaluating functional efficacy and immune resilience. Diabetic rodent transplantation models are routinely used to assess glycemic normalization and GSIS kinetics. Large animal models provide insight into scale-up feasibility, transplantation site optimization, and long-term durability.

Collectively, preclinical studies demonstrate that engineered islet-like cells can restore normoglycemia under immunodeficient or immunosuppressed conditions (Table 1). However, durable immune protection and scalable manufacturing remain active areas of investigation.

## 8. Clinical Translation of Engineered Stem Cell Therapies

### 8.1. Encapsulation-Based Trials

Encapsulation platforms, including those developed by ViaCyte (San Diego, CA, USA), Sernova (London, ON, USA), and Beta-O2 (Rosh HaAyin, Israel), have demonstrated safety and engraftment feasibility. While insulin independence has been variable, these studies validate device-based cell delivery strategies. Xenotransplantation of encapsulated swine islets has shown variable safety and effectiveness in clinical trials. In one trial, a collagen-covered islet encapsulation device enabled six out of twelve patients to reduce their insulin needs for four years. This device, which combined pig islets with Sertoli cells for protection, was implanted subcutaneously without the use of immunosuppressive medications [56]. ViaCyte’s Phase 1/2 clinical trial (VC-01; NCT04678557) assessed hPSC-derived pancreatic progenitor cells (PEC-01™) implanted subcutaneously in the Encaptra^®^ device (ViaCyte, Inc., San Diego, CA, USA). Consequently, ViaCyte launched a second Phase 1/2 study (VC-02; NCT03163511), utilizing PEC-01 cells in an open device to facilitate vascularization alongside systemic anti-inflammatory and immunosuppressive therapy (Table 2) [57,58].

To assess the safety and effectiveness of pancreatic endoderm cells (PECs) in establishing normoglycemia, PECs were strategically transplanted in T1D patients as part of a phase 1/2 clinical trial utilizing a non-immunoprotective macro-encapsulation technology. According to the one-year follow-up results, the initial cohort of 15 patients representing a single trial location got subcutaneous cell product transplantation in conjunction with an immunosuppressive regimen. Explanted grafts included mature β-cells that had been immunoreactive to insulin, IAPP, and MAFA. These results provide the first proof of meal-regulated insulin production by differentiated stem cells in humans [59].

Sernova created the Cell Pouch device, which features pre-vascularized polypropylene chambers for islet transplantation without the requirement for immunoprotection. In a 2012 experiment (NCT01652911), islets were implanted using vascularized pouches into three recipients who were simultaneously receiving immunosuppression. This resulted in a temporary increase in C-peptide levels (NCT03513939) [60,61,62].

The beta O2 βAir device, loaded with human islets, was placed subcutaneously in T1D patients (NCT02064309). While raising the total number of islets may improve their function, it is crucial to remember that the constant dependency on oxygen raises the possibility of infection, in spite of attempts to improve the longevity of encapsulated islets [63]. Semma Therapeutics (Cambridge, MA, USA)/Vertex (Boston, MA, USA) conducted clinical trials with differentiated stem cell-derived islet cell clusters. Semma developed these stem cells into two semipermeable polyvinylidene fluoride (PVDF) membranes that are intended for subcutaneous transplantation (NCT04786262) [64].

### 8.2. Fully Differentiated Stem Cell-Derived Islets

Fully differentiated stem cell-derived islet products have shown promising metabolic outcomes in early phase trials. Programs such as Vertex Pharmaceuticals shared positive results from Phase 1/2/3 clinical trials for Zimislecel (formerly VX-880), which is a type of pancreatic islet cell replacement therapy made from embryonic stem cells. This therapy is intended for people with type 1 diabetes who are receiving standard immunosuppression treatment. Recent studies are looking into the safety and effectiveness of Zimislecel for treating type 1 diabetes. In a phase 1-2 study, Zimislecel was given along with immunosuppressive therapy that does not include glucocorticoids, at different doses to 14 participants. Most of the participants, specifically 83% (which includes parts B and C, with 10 out of 12 participants), saw significant decreases in severe hypoglycemic episodes and became more independent from exogenous insulin over 12 months. All participants showed that the cells were successfully engrafted and began functioning like normal islet cells after receiving the treatment [65,66,67].

### 8.3. Gene-Edited Clinical Efforts

Emerging clinical strategies incorporate gene-edited or immune-modified cell lines designed to reduce rejection risk. CRISPR-modified and lentiviral-engineered platforms aim to improve graft persistence while minimizing systemic immunosuppression requirements. In 2025, researchers successfully transplanted genetically modified donor islet cells into a patient with long-standing type 1 diabetes, without the need for immunosuppressive therapy. The cells were genetically modified to prevent rejection using lentiviral transduction and clustered regularly interspaced short palindromic repeats (CRISPR)–CRISPR-associated protein 12b (Cas12b) editing technology. Twelve weeks after transplantation, the recipient had no immunological reaction against the gene-edited cells and had not been administered any immunosuppressive medications [68].

### 8.4. Food and Drug Administration-Approved Islet Cell Therapy

The US FDA approved Lantidra (Donislecel-jujn), the first single-donor-derived human pancreatic islet cellular suspension for infusion, targeting individuals with T1D experiencing inadequate insulin management and hypoglycemia unawareness. Manufactured by CellTrans Inc (Chicago, IL, USA)., its approval is based on Phase I/II studies showing graft survival in all ten patients, with 60% achieving insulin independence after five years. A Phase III trial revealed 21 patients had no hypoglycemia and maintained HbA1c < 6.5% over one year. Common side effects included nausea and fatigue, while some required discontinuation of immunosuppressive treatment, affecting islet functionality. This approval marks progress in T1D care amidst ongoing discussions about the classification of islets as medications rather than mini-organs, which may impact patient access [69,70,71,72].

## 9. Challenges and Limitations

Despite encouraging progress, several barriers must be addressed:

1. Tumorigenicity and safety. Residual undifferentiated cells and long-term oncologic risk remain concerns.
2. Scale and manufacturing. GMP-compliant large-scale production with consistent differentiation efficiency remains complex.
3. Immune rejection and autoimmunity. Achieving durable immune protection without lifelong systemic immunosuppression is a central objective.
4. Cost and accessibility. Manufacturing complexity and immunosuppression requirements may limit broad clinical accessibility. Addressing these domains will determine the scalability of engineered islet replacement.

### 9.1. Tumorigenicity and Safety Concerns

Tumorigenicity remains a major safety concern in the clinical application of stem cell-based therapies. Pluripotent stem cells (PSCs), including embryonic stem cells (ESCs) and induced pluripotent stem cells (iPSCs), possess extensive proliferative and differentiation capacity, which increases the risk that residual undifferentiated cells may form teratomas or other ectopic tissues following transplantation. If differentiation is incomplete prior to implantation, PSCs may generate tissues unrelated to the intended transplant site, posing significant clinical risks. Because stem cell-derived islet therapies involve long-term engraftment of proliferative cells, careful evaluation of tumorigenic potential and stringent purification of differentiated β-cell populations are essential prerequisites for clinical use [73,74].

### 9.2. Strategies to Ensure Safety

Several strategies have been developed to mitigate tumorigenicity and enhance the safety of stem cell-derived islet products. Controlled, stage-wise differentiation protocols are designed to efficiently direct PSCs toward pancreatic endocrine lineages while minimizing the persistence of undifferentiated cells. Sensitive quality control assays are increasingly implemented to detect rare pluripotent contaminants before transplantation. In parallel, genetic safeguards such as inducible “suicide switches” or drug-activated kill genes have been proposed to allow selective elimination of transplanted cells in the event of uncontrolled proliferation. Improvements in stem cell culture conditions and differentiation technologies have further enhanced the generation of functional insulin-producing β-cells while reducing pluripotent cell contamination. Despite these advances, long-term post-transplant surveillance remains essential to monitor potential tumor formation or ectopic tissue development [75].

### 9.3. Scale-Up and Manufacturing

The large-scale production of stem cell-derived β-cells represents a major translational challenge. Generating clinically relevant numbers of functional cells requires complex, multi-step differentiation protocols and tightly controlled culture conditions. Stem cells must be maintained in specialized media and environmental conditions to preserve their developmental potential and promote lineage-specific differentiation. Advanced culture systems, including three-dimensional platforms and bioreactors, are increasingly being explored to support efficient expansion and maturation of cells. However, ensuring consistent cell identity, purity, potency, and viability during large-scale manufacturing remains difficult, highlighting the need for standardized production processes and optimized bioprocessing technologies [76,77].

### 9.4. Challenges in Large-Scale Production

Despite significant progress in stem cell differentiation technologies, scaling up production for clinical applications remains complex. The cultivation of PSCs requires carefully controlled conditions, specialized media, and strict monitoring to maintain cell quality and functionality. Multi-stage differentiation into insulin-producing β-cells further increases production complexity, as each stage requires precise regulation of signaling pathways and culture environments. Achieving reproducibility at industrial scale is therefore challenging, particularly when large quantities of homogeneous, functional cells are required. Additionally, stringent quality control measures must be implemented to ensure uniformity in cell populations and prevent contamination with undifferentiated cells. These technical and logistical challenges often make large-scale production resource-intensive and may limit the rapid clinical translation of stem cell-based therapies [76,77].

### 9.5. Regulatory Hurdles

Regulatory approval of stem cell-derived therapies requires rigorous evaluation of safety, quality, and clinical efficacy. Agencies such as the U.S. Food and Drug Administration (FDA) and the European Medicines Agency (EMA) have established frameworks for advanced cellular therapies; however, regulatory pathways for complex precursor cell-based products are still evolving. Compliance with stringent chemistry, manufacturing, and controls (CMC) requirements is essential. Robust release criteria must be defined to assess product quality and functional potency, including assays for glucose-stimulated insulin secretion, evaluation of cellular composition, detection of residual pluripotent cells, and verification of genetic stability. For gene-edited or hypoimmunogenic cell lines, additional regulatory expectations include comprehensive characterization of genome edits, assessment of potential off-target effects, and implementation of long-term monitoring strategies for delayed adverse events [78].

### 9.6. Cost and Accessibility

The high cost associated with stem cell-derived therapies remain a significant barrier to their widespread clinical implementation. Manufacturing processes involve multi-stage differentiation protocols, specialized infrastructure, and extensive quality control testing, all of which contribute to substantial production costs. Additional expenses may arise from specialized transplantation procedures, long-term clinical monitoring, and the potential need for immunosuppressive therapy or implantable delivery devices. These economic factors may limit the availability of stem cell-based therapies unless scalable and cost-efficient production methods are developed.

### 9.7. Ensuring Accessibility to Patients

Improving patient access to stem cell-derived therapies will require advances in manufacturing scalability, cost reduction, and healthcare integration. Approaches such as automated bioprocessing systems, standardized manufacturing platforms, and the development of allogeneic “off-the-shelf” stem cell products may help reduce production costs and increase scalability. In addition, collaboration among academic institutions, industry partners, healthcare providers, and regulatory agencies will be critical to establish sustainable manufacturing pipelines and reimbursement strategies. Expanding clinical infrastructure and integrating stem cell therapies into healthcare systems will further support broader patient access. Ultimately, balancing technological innovation with affordability and regulatory compliance will determine whether stem cell-derived islet replacement therapies can become widely accessible treatments for diabetes.

## 10. Future Directions

Future progress will likely depend on convergence across multiple engineering domains:

- Development of universal donor stem cell lines.
- Immune stealth and hypoimmunogenic engineering.
- Biomaterial-integrated vascularizing scaffolds.
- Autologous iPSC and adult progenitor-based strategies.
- Precision medicine approaches tailored to disease subtype.

The field is transitioning from proof-of-concept differentiation toward integrated, systems-level engineering of durable, immune-resilient endocrine grafts. Continued interdisciplinary collaboration will be essential to translate these advances into scalable clinical therapies.

### 10.1. Next-Generation Stem Cell Technologies

#### 10.1.1. Advances in Genetic Editing and Synthetic Biology

Using genetic editing tools, like CRISPR-Cas9, to alter stem cells for therapeutic purposes is one of the most intriguing developments in stem cell research. With the use of this technology, scientists may precisely alter the genome, producing stem cells with certain genetic characteristics that increase their potential for therapeutic use. Gene editing, for instance, might be used to improve the differentiation of stem cells into specialized cell types, such as insulin-producing β-cells, or to fix genetic flaws in patient-specific stem cells. Additionally, methods from synthetic biology are being investigated to create stem cells with improved capabilities. To make stem cells better suited for therapeutic uses like the treatment of diabetes, for instance, synthetic gene circuits can be incorporated into them to give them the capacity to react to environmental conditions like variations in glucose levels [79].

#### 10.1.2. Development of Universal Donor Stem Cells

The creation of universal donor stem cells is another exhilarating field of stem cell research. These cells have undergone genetic modification to remove antigens that ordinarily cause recipients to mount an immunological defense. Allogeneic transplants using universal donor stem cells may lessen the requirement for immunosuppressive medications and increase the range of applications for stem cell treatments. At the heart of this endeavor are methods like gene editing and the utilization of iPSCs obtained from healthy donors. The resistance of universal donor stem cells to autoimmune response by the recipient needs to be addressed [80]. In the near future, universal donor or HLA-engineered allogeneic lines are the most plausible route to broad access, as they enable standardized, large-scale manufacturing.

### 10.2. Integration with Bioengineering and Biomaterials

#### Use of Smart Biomaterials for Islet Cell Delivery

The advancement of stem cell therapies heavily relies on bioengineering and biomaterials. Hydrogels and scaffolds are examples of smart biomaterials that are being developed to distribute stem cells to target organs in a controlled manner. To release cells in a regulated way, these materials can be designed to react to particular physiological stimuli, such as variations in pH or temperature. For instance, islet cells encapsulated in these materials can be sent to the pancreas for the treatment of diabetes, and the biological material also promotes the survival and functionality of the cells. The function of islets is preserved in the presence of the three-dimensional scaffolds [81].

### 10.3. Personalized Medicine Approaches

#### 10.3.1. Tailoring Therapies Based on Patient-Specific Factors

The future of stem cell-based treatments depends heavily on personalized medicine techniques. It is feasible to optimize therapeutic efficacy and reduce potential negative effects by customizing therapies for each patient. Using patient-specific iPSCs made from their own tissues, which can then develop into the appropriate cell types for transplantation, is one example of a personalized method. This might ensure that the cells being transplanted are the most appropriate for the patient’s particular requirements while preventing immunological rejection [82].

#### 10.3.2. Use of Patient-Derived Induced Pluripotent Stem Cells

There are several benefits to using patient-derived iPSCs in individualized therapy. Researchers can simulate diseases in vitro and determine the best treatment strategies using these cells, which are produced by reprogramming a patient’s somatic cells. Personalized therapies that target the root causes of illness at the cellular level can be created by employing iPSCs from individuals with certain genetic disorders [83]. Patient-derived iPSCs will likely remain most relevant for select indications such as monogenic diabetes, complex immunologic histories, or specific research and personalized disease modelling applications rather than for routine cell replacement therapy in the general T1D population.

## 11. Summary and Conclusions

The pursuit of stem cell-based islet replacement has evolved from early differentiation experiments to a multidisciplinary engineering endeavor integrating developmental biology, genome editing, biomaterials science, and translational immunology. Significant progress has been made in generating insulin-producing cells with increasingly robust glucose responsiveness, validating the feasibility of restoring functional β-cell mass through engineered platforms.

PSC-derived islets provide scalability and manufacturing consistency, supporting standardized clinical deployment. In parallel, adult pancreatic progenitor-based strategies offer a lineage-restricted alternative that may leverage intrinsic endocrine programming while potentially mitigating tumorigenic concerns associated with pluripotency. These approaches should not be viewed as competing paradigms but as complementary engineering solutions aimed at achieving durable endocrine restoration.

Despite substantial advances, durable immune protection, complete functional maturation, scalable GMP production, and broad accessibility remain central challenges. Continued refinement of immune-stealth engineering, encapsulation technologies, optimized transplantation sites, and physiologic maturation strategies will be essential to achieving sustained insulin independence without excessive systemic immunosuppression.

The field now stands at a pivotal stage in which proof of concept has transitioned into early clinical validation. The continued convergence of stem cell biology and precision bioengineering holds the potential to redefine treatment paradigms for insulin-dependent diabetes, moving beyond exogenous insulin replacement toward restoration of coordinated, physiologic islet function.

## Figures and Tables

**Table 1:** Preclinical studies investigating engineered stem cell-based strategies for pancreatic islet replacement in diabetes models.

Stem Cell Source/Model	Experimental Strategy	Key Findings	Reference
Stem cell-derived β-cells	Transplantation into immune-competent animals	Achieved restoration of glycemic control following transplantation of SC-derived β-cells.	[41]
Alginate-encapsulated SC-β-cells	Implantation in STZ-induced diabetic C57BL/6J mice	Maintained glucose regulation for 174 days with viable insulin-producing cells and minimal fibrosis.	[42]
Encapsulated islets	Transplantation into epididymal or mammary fat pads of diabetic mice	Demonstrated long-term graft survival exceeding 100 days.	[43]
hPSC-derived pancreatic progenitor cells	Macroencapsulation using TheraCyte device	Generated glucose-responsive insulin-producing cells and reversed diabetes in mice.	[44]
Stem cell-derived islet grafts	Pre-transplant vascularization strategies	Improved graft vascularization, glucose sensing, insulin delivery, and graft longevity.	[30]
Islets co-transplanted with HUCPVC-MSCs	Co-transplantation approach in mice	Increased graft vascularization and improved glycemic regulation compared with islet transplantation alone.	[45]
Donor islets with angiogenic stimulation	Hydrogels containing angiogenic factors and VEGF-A gene expression	Enhanced islet survival, growth, vascularization, and functional performance.	[46,47,48]
Porcine islet xenografts	Anti-CD40 (2C10R4) monoclonal antibody with tacrolimus-based immunosuppression	Prevented xenograft rejection in diabetic rhesus monkeys; median graft survival for 60 days.	[49]
Islet allotransplantation	Anti-CD40L monoclonal antibody (AT-1501) therapy	Improved long-term transplant retention and increased C-peptide levels in cynomolgus macaques.	[50]
hPSC-derived SC-β-cells	Cytoskeleton modulation using latrunculin A during differentiation	Generated functional β-cells with biphasic glucose-stimulated insulin secretion; restored normoglycemia in mice for up to 9 months.	[51]
iPSC-derived islet organoids	Transplantation into brown adipose tissue (BAT)	BAT provided a vascularized niche supporting organoid survival and improved function.	[52]
Genetically engineered stem cell-derived β-like cells	HLA class I deletion and inducible PD-L1 overexpression	Reduced activation of diabetogenic CD8^+^ T cells and improved immune protection.	[53]
Hypoimmunogenic engineered hPSCs	Deletion of polymorphic HLAs while retaining HLA-A2 and HLA-E/F/G	Reduced risk of alloimmune rejection and improved compatibility with recipients.	[32]
Stem-cell-derived islets	Single-cell RNA sequencing after transplantation	Transplanted SC-islet cells acquired mature β-cell gene expression (e.g., MAFA, G6PC2).	[54]
Genetically modified SC-β-cells	Cytokine secretion (IL-10, TGF-β, modified IL-2) to recruit Tregs	Created a tolerogenic microenvironment preventing rejection and treating diabetes in NOD mice for ~8 weeks.	[31]
A2-CAR-T cell model	HLA-A2-specific CAR-T cells targeting transplanted islets	Enabled investigation of alloimmune rejection and immune-evasion strategies in transplantation models.	[55]

**Table 2:** Summary of key clinical programs in islet and stem cell-derived islet replacement, highlighting how differences in cell source, delivery strategy, and immune protection translate into distinct regulatory trajectories and clinical outcomes.

Program/Product	Cell Source	Delivery Strategy	Immune Protection	Trial Phase/Status	Main Reported Outcomes
Lantidra (donislecel)	Allogeneic donor islets (cadaveric pancreas)	Intraportal infusion into liver	Systemic immunosuppression (standard islet transplant regimens)	FDA-approved for select adults with T1D and severe hypoglycemia	Improved glycemic control and reduced severe hypoglycemia; variable insulin independence; risks from chronic immunosuppression and limited donor supply.
VX-880 (zimislecel)	Allogeneic PSC-derived islet cells	Intraportal hepatic infusion	Systemic immunosuppression	Phase 1/2; phase 3 program initiated	Marked HbA1c and time-in-range improvement; many participants achieving insulin independence; safety acceptable so far but long-term durability and tumorigenicity still under evaluation.
Encapsulation device A (ViaCyte,PEC-Encap)	PSC-derived pancreatic progenitors	Subcutaneous macroencapsulation device	Local device-based immune isolation; minimal or no systemic immunosuppression	Early-phase clinical trials (mixed results; some programs discontinued or modified)	Demonstrated safety and feasibility of device implantation; limited or inconsistent insulin production due to fibrosis and poor vascularization in many subjects.
Encapsulation device B (Sernova Cell Pouch with islets)	Allogeneic donor islets (iPSC-derived islets)	Vascularizing subcutaneous pouch device	Combination of device protection plus systemic immunosuppression (in current trials)	Phase 1/2	Feasible engraftment in pre-vascularized pouches; improvements in C-peptide and glycemic control in some patients; device fibrosis and need for systemic immunosuppression remain issues.
Gene-edited/hypoimmune PSC-islets	Gene-edited PSC-derived islets (HLA-modified, immune-stealth)	Typically, intraportal or device-based delivery	Intrinsic immune evasion via gene editing; goal of reduced/no systemic immunosuppression	Preclinical to early phase 1	Prolonged survival and function in immunocompetent animal models; early human safety/engraftment data pending; long-term safety and immune escape risk unknown.
Autologous iPSC-derived islets	Patient-specific iPSCs or CiPSCs	Extra-hepatic implantation (muscle or abdominal wall)	Autologous grafts; may still use systemic immunosuppression in early studies	Early single-patient or small pilot studies	Restoration of endogenous insulin production and insulin independence in isolated cases; workflows complex, costly, and not yet scalable; true immunosuppression-free durability not established.

## Data Availability

No new data were created or analyzed in this study.
